# Physical exercise associated with NO production: signaling pathways and significance in health and disease

**DOI:** 10.3389/fcell.2015.00019

**Published:** 2015-04-02

**Authors:** Elena Y. Dyakova, Leonid V. Kapilevich, Victor G. Shylko, Sergey V. Popov, Yana Anfinogenova

**Affiliations:** ^1^Department of Sporting Health Tourism, Physiology, and Medicine, National Research Tomsk State UniversityTomsk, Russia; ^2^Institute of Physics and Technology, National Research Tomsk Polytechnic UniversityTomsk, Russia; ^3^Federal State Budgetary Scientific Institution “Research Institute for Cardiology,”Tomsk, Russia

**Keywords:** physical exersize, skeletal muscle, nitric oxide, physiology, myokine

## Abstract

Here we review available data on nitric oxide (NO)-mediated signaling in skeletal muscle during physical exercise. Nitric oxide modulates skeletal myocyte function, hormone regulation, and local microcirculation. Nitric oxide underlies the therapeutic effects of physical activity whereas the pharmacological modulators of NO-mediated signaling are the promising therapeutic agents in different diseases. Nitric oxide production increases in skeletal muscle in response to physical activity. This molecule can alter energy supply in skeletal muscle through hormonal modulation. Mitochondria in skeletal muscle tissue are highly abundant and play a pivotal role in metabolism. Considering NO a plausible regulator of mitochondrial biogenesis that directly affects cellular respiration, we discuss the mechanisms of NO-induced mitochondrial biogenesis in the skeletal muscle cells. We also review available data on myokines, the molecules that are expressed and released by the muscle fibers and exert autocrine, paracrine and/or endocrine effects. The article suggests the presence of putative interplay between NO-mediated signaling and myokines in skeletal muscle. Data demonstrate an important role of NO in various diseases and suggest that physical training may improve health of patients with diabetes, chronic heart failure, and even degenerative muscle diseases. We conclude that NO-associated signaling represents a promising target for the treatment of various diseases and for the achievement of better athletic performance.

## Introduction

Nitric oxide (NO) was first discovered as a marker of inflammation and a regulator of vascular tone. However, the following studies demonstrated that spectrum of the regulatory functions of this molecule is much broader. Indeed, NO production changes in response to various stimuli in health and disease.

One of the intriguing facts is an increase in NO production in skeletal muscle in response to physical exercise. Previously, the role of NO in skeletal muscle was discussed in a comprehensive review written by Stamler and Meissner (2001). A brief review of this subject was published by Reid (1998) with emphasis on synthesis, distribution and functional importance of NO in skeletal muscle. Maréchal and Gailly divided the effects of NO in skeletal muscle into two groups, namely: direct and cGMP-mediated effects (Maréchal and Gailly, 1999). Kingwell discussed NO-mediated metabolic regulation during exercise in health and disease (Kingwell, 2000a) including cardiovascular pathologies (Kingwell, 2000a). Bredt (1999) and Kingwell (2000b) presented a literature review covering a wide range of questions regarding the NO effects on the organism. Kaminski and Andrade (2001) also reviewed NO-mediated biological effects on the muscle and their role in muscle disease.

The subject of NO production in physical exercise has been of interest for many years; new studies brought more clarity to the overall picture of the role of NO. However, some aspects of this research area remain controversial. Indeed, the results of some studies suggest that eNOS and nNOS are not critical for the exercise-induced augmentation of mitochondrial biogenesis in skeletal muscle (Wadley et al., 2007) whereas other studies provide evidence that exercise and mitochondrial biogenesis are associated with NO-dependent changes in AMPK and PGC-1α (Lira et al., 2010).

The present review focuses on recent data and highlights the latest advances in studying the intrinsic interplay between NO and physical exercise. The manuscript reviews NO-mediated effects on skeletal myocytes, hormone regulation, and local microcirculation. The authors discuss NO-dependent signaling pathways and significance of the exercise-induced NO production in health and disease.

### Nitric oxide synthases (NOS) in skeletal muscle

Nitric oxide is generated from amino acid L-arginine by NOS (Moncada and Higgs, 1993). The second significant source of NO refers to its bound forms. According to estimates, 70–90% of NO is stored in S-nitrosothiols, main source of NO in tissues (Nijkamp and Folkerts, 1995).

Three isoforms of NOS have been identified: an endothelial isoform (eNOS; gene located on chromosome 7), a neuronal isoform (nNOS; gene located on chromosome 12) and a macrophage or inducible isoform (iNOS; gene located on chromosome 17). eNOS and nNOS are the normal constituents of healthy cells. They both contribute to physiological regulation (Lugg et al., 1995). By using immunocytochemical methods, Kobzik et al., demonstrated that nNOS (Kobzik et al., 1994) and eNOS (Kobzik et al., 1995) are expressed in skeletal muscle. Moderate exercise increases activity of the entire NOS pool (i.e., the cumulative activity of eNOS, nNOS, and iNOS). Electrical stimulation of skeletal muscular cells is associated with the increased iNOS content, NO production, and NF-kB activation (Lambertucci et al., 2012). A single moderate exercise increases NO content through an activation of nuclear factor kappa B (da Silva Rossato et al., 2014). nNOS expression is significantly higher in mice deficient for eNOS gene (eNOS^−/−^) (Wadley et al., 2007). In mature skeletal and cardiac myocytes, nNOS contains a 34-amino-acid insert as a result of the alternative splicing of nNOS pre-RNA between exons 16 and 17 (Brenman et al., 1997). A skeletal muscle-specific nNOS is identified as a component of dystrophin complex (Chao et al., 1998).

There are three different fractions of nNOS: nNOSβ of Golgi apparatus, sarcolemmal nNOSμ, and cytoplasmic nNOSμ (Silvagno et al., 1996; Percival et al., 2010). To determine the location of cytoplasmic nNOS, Percival et al. immunolabeled single muscle fibers. Four pan-specific anti-nNOS antibodies raised against distinct epitopes detected that nNOS localize to sub-sarcolemmal puncta. The labeling pattern resembled that of the skeletal muscle Golgi complex. Moreover, nNOS colocalizes with GM130, a marker of the cis-face of the Golgi complex suggesting the existence of a previously unrecognized Golgi-associated nNOS compartment in the skeletal muscle cells. Evidence suggests that (i) loss of signaling from nNOSμ does not limit force production during or after exercise; (ii) nNOSβ is a critical regulator of skeletal muscle fatigue and postexercise force output; (iii) during exercise, nNOSμ signaling maintains blood delivery to the muscle, while nNOSβ regulates fatigue resistance of the muscle and postexercise force output; and (iv) the differential targeting of nNOS splice variants creates functionally distinct NO signaling microdomains, at which NOS-derived NO acts locally (Percival et al., 2010). The nNOS splice variant, localized to the Golgi complex, regulates microtubule cytoskeleton integrity in skeletal muscle (Percival et al., 2010). Sarcolemmal nNOSμ affects muscle ability to absorb oxygen and is recruited to the sarcolemma by binding to the dystrophin-associated protein α-syntrophin (Thomas et al., 2003). nNOSμ is responsible for maintaining blood supply during physical exercise, glucose homeostasis modulation, muscular mass control, and regulation of the fatigue resistance (Percival et al., 2010). Intense exercise that lasts for 10 days is sufficient to uniformly increase nNOSμ expression in untrained individuals (McConell et al., 2007).

### Reactive oxygen species limit nitric oxide signaling

Data suggest that NO content is decreased in the exhaled air from the volunteers after running on a treadmill at altitudes of 2800 and 180 m for several minutes. Partial pressure of NO significantly decreases at both altitudes (Stang et al., 2014). What are the factors limiting NO signaling? The bioavailability of NO depends on the balance between the enzymatic and non-enzymatic NO production and NO removal due to interactions between NO and reactive oxygen species (ROS). The presence of the latter depends on ROS formation by NADPH and xanthine oxidases in mitochondria. It also depends on ROS removal by antioxidant defense systems (Reutov and Sorokina, 1998; Reutov, 1999). When NO is produced enzymatically, L-arginine serves as a substrate for NOS (Moncada and Higgs, 1993). The second significant source of NO is associated with its bound forms. Cells generate NO from nitrates and nitrites either non-enzymatically or enzymatically by NO^−^_3_ and NO^−^_2_ reductases (Cantu-Medellin and Kelley, 2013) Some estimates suggest that 70–90% of NO is generated from S-nitrosothiols, main source of NO in tissues (Nijkamp and Folkerts, 1995). Nitric oxide exerts its regulatory effects through several mechanisms. The first mechanism consists in the S-nitrosylation where NO binds to the mixed-valence metals in metalloproteins, SH-groups in cysteine, and to tyrosine. Nitrosylation of proteins changes their biological properties and functions (Broillet, 1999; Feng, 2012). Other mechanisms are associated with NO-induced guanylate cyclase activation, cGMP increase, calcium signaling modification, and reverse protein phosphorylation (Maréchal and Gailly, 1999; Moyna and Thompson, 2004; Tschakovsky and Joyner, 2008).

Excess of ROS both reduces NO bioavailability and damages cells of the cardiovascular system. Physical activity significantly improves cardiovascular function, in particular, through the increased bioavailability of NO, enhanced endogenous antioxidant protection, and the reduced expression of ROS-forming enzymes (Gliemann et al., 2014). Besides, there are data that superoxide can directly suppress NO without affecting expression or activity of eNOS (Paolocci et al., 2001).

### Effects of NO modulators on skeletal muscle performance

Hemodynamic reserve largely limits physical performance. Nitric oxide can control the local hemodynamics in dependence on physical effort at the level of an individual muscular group. In this regard, the effects of NO donors on physical performance are of great interest.

Administration of L-arginine to healthy volunteers does not significantly change metabolic and hormonal parameters compared with the effects of physical exercise (Alvares et al., 2014). However, intake of L-arginine increases muscle strength (Alvares et al., 2014) and recovery after physical exercise (Santos et al., 2002). Indeed, single administration of L-arginine in healthy individuals before strength exercise increases the volume of blood in the muscle. It is interesting that no significant difference in plasma concentrations of nitrites after acute administration of L-arginine alone or in response to resistance exercise is reported (Alvares et al., 2012a,b). Beneficial effects of L-arginine are explained by dilation of blood vessels in response to NO increase.

Infusion of a non-specific NO synthase inhibitor, L-NMMA, significantly reduces glucose uptake in the leg due solely to a decrease in an arteriovenous glucose difference, as there is no effect of L-NMMA infusion on local blood flow during exercise. These data may suggest that NO production contributes substantially to an exercise-mediated skeletal muscle glucose uptake in humans independently of skeletal muscle blood flow (Bradley et al., 1999). L-arginine infusion during exercise also significantly increases skeletal muscle glucose clearance in humans. Because plasma insulin concentration is unaffected by L-arginine infusion, greater NO production may be responsible for this effect (McConell et al., 2006). Phosphodiesterase inhibitor, EMD360527, causes an increase in vasodilation in systemic and pulmonary circulation at rest and during treadmill exercise in pigs (Zhou et al., 2014a,b). Therefore, pharmacological support targeting NO-mediated signaling may be promising for improving athletic performance.

### Nitric oxide affects hormone regulation in skeletal muscle

Large body of evidence suggests that effects of NO are not limited by its impact on regional hemodynamics and microcirculation (Del Pozzi et al., 2013). Nitric oxide can alter energy supply in skeletal muscles through the modulation of hormones targeting skeletal muscle. Indeed, NO stimulates production of human growth hormone (GH) in cultured fetal pituitaries and GH-secreting adenomas (Rubinek et al., 2005). There is a hypothesis that L-arginine improves exercise performance by increasing NO production and the levels of insulin and growth hormone (GH). However, 4-week L-arginine supplementation does not affect metabolic and hormonal parameters beyond those changes achieved with the exercise alone (Campbell et al., 2006; Alvares et al., 2014). eNOS heterozygous mice have lower glucose levels during exercise compared with the wild-type mice whereas eNOS knockout mice demonstrate lower levels of glucose than the eNOS heterozygous mice and become hypoglycemic after the exercise (Lee-Young et al., 2010). These data suggest that eNOS is involved in the regulation of glucose metabolism in physical exercise. These NO effects are mediated through the changes in the insulin production. Quantitative reduction in eNOS protein expression can result in metabolic dysregulation *in vivo*. On the other hand, hormones can affect NOS. For example, treatment of cultured human endothelial cells with somatotropin results in significant increase in the eNOS gene and protein expression and NO release whereas production of intracellular ROS is significantly reduced in the presence of somatotropin. Enhanced eNOS gene/protein expression correlates well with the enzyme activity (Thum et al., 2003). It is important, that vascular and hormonal mechanisms of NO effects on the skeletal muscular contractions cannot be studied separately from each other.

### Direct effects of NO on skeletal myocytes

Nitric oxide indirectly modulates supply of the skeletal myocytes with oxygen and energy substrates via the regulation of blood flow and hormones (Santos et al., 2002; Alvares et al., 2012a, 2014). However, there is evidence that NO can directly affect skeletal muscle through the modulation of energy metabolism. In general, the effects of NO are mediated through cGMP that, in turn, is metabolized by phosphodiesterases. An inhibitor of phosphodiesterase 5, sildenafil, increases protein synthesis, changes protein expression and nitrosylation, and decreases fatigability of human skeletal muscle (Sheffield-Moore et al., 2013). In knockout mouse model, eNOS affects the ATP levels. Basal ATP levels are distinct in skeletal muscles in mice with wild-type (WT), partial expression (±), or no expression (-/-) of eNOS. Uptakes of glucose and long-chain fatty acid as well as glycogenolysis during exercise are markedly accelerated in liver tissue and skeletal muscle in the eNOS^−/−^ mice compared with eNOS^±^ and WT mice (Lee-Young et al., 2010).

### Nitric oxide and mitochondria

In skeletal muscle, mitochondria are highly abundant and play a pivotal role in metabolism (Hood et al., 2006). Nitric oxide is a plausible regulator of mitochondrial biogenesis and can directly affect respiration in myocytes. nNOSβ isoform, associated with Golgi apparatus, affects mitochondrial function. Indeed, long-term exposure to NO triggers mitochondrial biogenesis in mammalian cells and tissues through the activation of guanylate cyclase and generation of cGMP. The NO/cGMP-dependent mitochondrial biogenesis is associated with enhanced coupled respiration and ATP content in skeletal muscle (Nisoli et al., 2004). Nitric oxide induces mitochondrial biogenesis in skeletal myocytes through upregulation of the peroxisome proliferator-activated receptor-γ coactivator 1α (PGC-1α) (Scarpulla, 2008). eNOS and nNOS are differentially involved in the basal regulation of mitochondrial biogenesis in skeletal muscle though Wadley et al. belives they are not critical for the exercise-induced increases in mitochondrial biogenesis in this tissue (Wadley et al., 2007). The question on how critically NOS affects metabolic function in skeletal muscle remains controversial. Nitric oxide interacts with the metabolic sensor enzyme, AMPK (Lira et al., 2007). Nitric oxide and AMPK cooperatively regulate PGC-1α in skeletal muscle cells. The AMPKα1 isoform is identified as a mediator of NO-induced effects in skeletal muscle cells. The authors propose a model of synergistic interaction between AMPK and NOS for maintenance of metabolic function in skeletal muscle (Lira et al., 2010). These authors consider this interaction critical.

A phenotype of mice with quantitative reductions in eNOS expression (approximately 40%) is characterized by reduced expression of mitochondrial oxidative phosphorylation complex, impaired ATP level, and increased total NOS activity and CaMKII phosphorylation in skeletal muscle. A 30-min bout of acute exercise *in vivo* elicits a number of physiological processes that depend on eNOS expression with the exercise-induced AMPKα phosphorylation being reduced in parallel with decrease in eNOS expression. Ablation of eNOS results in impaired exercise capacity, hypoglycemia, and increased plasma lactate levels without changing the mitochondrial content (Lee-Young et al., 2010). However, effects of NO are rarely unidirectional and ultimately beneficial. Indeed, NO regulates mitochondrial oxygen consumption through competitive binding to cytochrome-c oxidase resulting in increased free radical content and mitochondrial fragmentation.

NO exerts an inhibitory effect on the electron transport chain. Low physiological NO concentration reversibly inhibits mitochondrial oxygen consumption by binding to a3-heme-site of cytochrome oxidase in competition with oxygen. Increased NO concentrations result in peroxinitrite production that, in turn, irreversibly inhibits complex I and II of the electron transport chain. Blood flow and oxygen consumption during exercise can be regulated both at the level of the vasculature and in the adjacent muscle mitochondria by similar parallel signals (Boushel et al., 2012).

NOS inhibition potentiates mitochondrial respiration (Boushel et al., 2012) as demonstrated in experiments where L-NMMA was infused in femoral artery.

Characteristic property of NO is the ability to reversibly and competitively inhibit cytochrome c oxidase, the terminal enzyme of the mitochondrial respiratory chain (Bolaños et al., 1994; Brown and Cooper, 1994; Cleeter et al., 1994). Inhibition of NOS is associated with increased oxygen consumption in canine skeletal muscles at rest (Shen et al., 1994). Physiological NO levels (about 20 nM) indeed affect oxygen consumption in tissues (Thomas et al., 2004). The O_2_/NO ratio of about 500 is necessary to achieve 50%-inhibition of cytochrome c oxidase (Boveris et al., 2000). Binding of NO to cytochrome c oxidase triggers intracellular signaling events including diversion of oxygen to non-respiratory substrates and ROS generation with potentially hazardous consequences (Erusalimsky and Moncada, 2007). Considering that NO production by NOSs is oxygen-dependent in normoxic tissues and is limited in hypoxia, nitrate-nitrite-NO pathway is significantly facilitated and, therefore, can complement NOS-based NO production in conditions that occur during exercise or ischemia (van Faassen et al., 2009). Mitochondrial enzyme content and activity are increased in skeletal muscle after the period of exercise (Little et al., 2011). Nitrates reduce oxygen cost of exercise (Larsen et al., 2012).

Bailey et al., used ^31^P magnetic resonance spectroscopy to evaluate metabolic abnormalities in skeletal muscle during knee-extensor exercise in humans. Data showed that reduction in phosphocreatine concentration was attenuated and ATP turnover rate was lower during exercise in the presence of dietary nitrate supplementation compared with placebo (Bailey et al., 2010). These results show that nitrate exerts its effects on the targets beyond mitochondria as well, presumably on the contractile actin-myosin filaments or sarcoplasmic reticulum calcium load (Larsen et al., 2012). Nitric oxide also stimulates mitochondrial fragmentation, which can be caused by too much mitochondrial fission or mitochondrial fusion inhibition causing bioenergetic failure (Knott and Bossy-Wetzel, 2010).

eNOS expression, mitochondrial biogenesis, mitochondrial volume density and number, and both basal and insulin-stimulated levels of glucose uptake are increased in the left ventricle of wild type mice subjected to swimming exercise. In contrast, genetic deletion of eNOS prevents all these adaptive phenomena (Vettor et al., 2014).

This evidence suggests that mitochondria may represent a target for NO that is released during exercise.

### Myokines and NO-mediated signaling

Myokines comprise cytokines and other molecules that are expressed and released by muscle fibers and exert autocrine, paracrine and/or endocrine effects. Hundreds of myokines have been identified, but information regarding myokine-dependent regulation triggered by contraction and other stimuli is lacking in most cases (Eckardt et al., 2014). There is lack of data about putative interplay between NO-mediated signaling and myokines. However, sparse evidence suggests that such interplay can in fact exist (Takahashi et al., 1999; Figueras et al., 2004; Steensberg et al., 2007; Ouchi et al., 2008; Murata et al., 2012; Sandonà et al., 2012; Baum et al., 2013; Most et al., 2013; Chong et al., 2014).

For example, studies demonstrate interplay between interleukin myokines and NO-signaling (Takahashi et al., 1999; Steensberg et al., 2007; Murata et al., 2012; Chong et al., 2014). Steensberg with coworkers hypothesized that exercise-induced NO production is an important signaling event for expression of myokines IL-6 and IL-8 in the muscle. L-NAME causes significant reductions in the exercise-induced mRNAs expression of these myokines. Nitroglycerin infusion significantly augments expression of mRNAs attenuated by L-NAME. These findings confirm an idea that NO production is involved in the regulation of myokine gene expression in muscle during exercise (Steensberg et al., 2007). Association between nitrite and myokine IL-6 levels is shown in crush syndrome (CS) caused by muscle compression of the limb with subsequent reperfusion. Tissue nitrite level in injured muscles is significantly reduced in the CS group compared with the sham group during ischemia/reperfusion injury. Nitrite administration to CS animals restores NO bioavailability by enhancing nitrite levels in the muscle, causing a reduction of rhabdomyolysis markers such as potassium, lactate dehydrogenase, and creatine phosphokinase. Nitrite treatment also reduces plasma levels of IL-6 and myeloperoxidase activities in the muscle and lung tissues. Murata with coauthors reported the therapeutic effects of nitrite in CS (Murata et al., 2012). IL-13 prevents the deterioration of contraction induced by endotoxin through inhibition of NO production mainly in the type I muscle fibers in a septic animal model (Takahashi et al., 1999). In skeletal muscle of tumor-bearing animals, administration of myokine IL-15 modulates apoptosis and decreases iNOS protein expression by 73%, suggesting that NO formation and muscle apoptosis may be related. IL-15 seems to reduce or suppress protein loss and apoptosis associated with muscle wasting in experimental model (Figueras et al., 2004).

In non-muscular tissue, myokine protein, angiopoietin-like 4 (ANGPTL4), accelerates wound healing. Exogenous ANGPTL4 modulates several regulatory networks involved in the cell migration, angiogenesis, and inflammation. Signaling pathways involving ANGPTL4 are not clear, but, in wound epithelia, ANGPTL4 induces NO production through upregulation of iNOS expression (Chong et al., 2014). Ouchi with coworkers investigated the role of Follistatin-like 1 (Fstl1) in the regulation of endothelial cell function and blood vessel growth in the muscle. Fstl1 is a myokine that promotes endothelial cell function. It stimulates ischemia-triggered revascularization though the activation of Akt-eNOS signaling. Increased expression of Akt1 in skeletal muscle increases muscle capillary density, myofiber growth, Fstl1 induction, and flow recovery associated with an increase in eNOS phosphorylation in the ischemic hind limbs of wild-type mice. The stimulatory effect of Ad-Fstl1 on ischemic limb reperfusion is abolished in mice lacking eNOS (Ouchi et al., 2008).

Animals subjected to skeletal muscle ischemia/reperfusion have significantly lower NO levels in muscle tissue when administered with intravenous myokine vascular endothelial growth factor (VEGF) compared with control animals subjected only to ischemia/reperfusion (Kirisci et al., 2013). The mRNA levels of VEGF-A are lower in the muscle of nNOS-knockout mice compared with the wild-type littermates (Baum et al., 2013). Unresolved ischemia entails excessive VEGF accumulation with aggravated VEGF receptor-2 degradation and blunted *in vivo* signaling through the proangiogenic phosphoinositide-3-kinase/Akt/eNOS cascade in mice (Most et al., 2013).

More research is needed to identify detailed mechanisms of the interactions between NO-mediated signaling and myokines in skeletal muscle during physical exercise.

### Nitric oxide is an important factor in therapeutic effects of physical activity in various diseases

Physical activity is currently considered as an important therapeutic factor in various diseases. To successfully combine the benefits of exercise with pharmacotherapeutic approaches, one must have a clear understanding of how exercise affects patient's body. Treatment of diabetes is one of the best examples in this regard.

#### Physical exercise and NO in diabetes mellitus

On one hand, the presence of glucose tolerance in diabetic skeletal muscle raises concerns about relevance of physical exercise for correction of diabetes symptoms. However, both experimental and clinical data suggest that beneficial effects of exercise are directly associated with NO production. The course of 40-min exercise 3 days per week for 12 weeks results in decreased plasma concentrations of NO and endothelin 1 in patients with impaired glucose tolerance (Kasimay et al., 2010). It may be that the decrease in endothelin 1 plays a primary role in mediating beneficial effects of exercise in this case as NO does not directly affect glucose utilization.

In rats with type 1 diabetes mellitus, eNOS protein expression increases in sedentary lifestyle and further increases when animals are subjected to exercise. The authors conclude that exercise alleviates impaired eNOS- and nNOS-dependent arteriolar responses in type 1 diabetes mellitus without changing nNOS in brain tissue (Mayhan et al., 2011). In diabetic rats, exercise training attenuates subcortical infarct volume after ischemia-reperfusion of the middle cerebral artery (Arrick et al., 2012).

The endurance training in rats with type 1 diabetes mellitus is more effective than resistance training. It improves acetylcholine-induced vasorelaxation responsiveness (Murias et al., 2013).

The study by Rodrigues with coworkers showed that exercise improves glycemic control, increases NO bioavailability, better controls oxidative stress, and corrects renal function. In this study, diabetic nephropathy was simulated in Wistar rats subjected to 8-week treadmill training with a work rate of 16 m/min, 60 min/d for 5 days per week. Diabetic animals had reduced creatinine and urea clearance and NO excretion and increased blood glucose concentrations, albuminuria, and excretion of the thiobarbituric acid reactive substances compared with control (Rodrigues et al., 2011). Additionally, 3- to 4-week exercise training corrected erectile dysfunction caused by central administration of exogenous NO donor, sodium nitroprusside, in rats with type 1 diabetes mellitus (Rodrigues et al., 2011; Zheng et al., 2011).

Type 2 diabetic patients have elevated levels of systemic free radicals severely restricting the endothelial NO bioavailability and contributing to endothelial dysfunction. Physical exercise enhances NO synthesis in blood vessels through increases in eNOS availability and activity (Brinkmann et al., 2011). In patients with type 2 diabetes, 6-week training (90-min exercises 4 times per week) partially restores NOS activity in the erythrocytes (Ladage et al., 2012).

Functional evaluation of the isolated aortic rings showed that exercise training restores acetylcholine-induces endothelial-dependent vasodilation in mice with type 2 diabetes mellitus without increasing eNOS protein expression. This improvement is associated with an activation of the adiponectin-dependent and independent pathways (Lee et al., 2011; Cordani et al., 2014).

Overall, many authors have reported changes in NOS activity in diabetes. However, only a few of them attempted to study therapeutic modulation of NO production in this condition.

#### Physical exercise in cardiovascular diseases

12-week course of Tai Chi exercise results in an increase of NO content in blood plasma of patients with essential hypertension. This effect is not observed in the control group of healthy volunteers and untrained hypertonic patients (Pan et al., 2014). The experiments in laboratory animals show that 5-week training improves myocardial antioxidant capacity, prevents excessive NO synthesis, and limits formation of cytotoxic peroxynitrite in the reaction between NO and O_2_ (Farah et al., 2013).

Both aerobic and resistance training increases peak oxygen, improves cardiac function, reduces mortality, and enhances performance and the quality of life in patients with chronic heart failure. These improvements occur due to the effects of training on the peripheral circulation and skeletal muscle, rather than on the heart itself. Exercise training and effort promotes the synthesis and release of nitric oxide, decreases oxidative stress, increases blood flow in active skeletal muscle, improves skeletal muscle metabolism, improves angiogenesis, reduces sympathetic arousal, and increases parasympathetic arousal that alleviates cardiac dysrythmia and ischemia (Gąsiorowski and Dutkiewicz, 2013).

Chronic heart failure interferes with NO-mediated regulation of oxygen delivery-utilization matching such that microvascular oxygenation falls faster in response to increases in metabolic demand in skeletal muscle. Physical exercise training improves kinetics of oxygen partial pressure by NO-dependent mechanisms in microvessels after muscle contractions in healthy young individuals. The model of murine chronic heart failure shows that there are two pathways that improve oxygenation in skeletal muscle microvessels and one of them is NO-mediated (Hirai et al., 2014).

#### Nitric oxide in skeletal muscle diseases

Abnormal NO-signaling occurs in degenerative muscle diseases including muscular atrophies and dystrophies (Wang and Lu, 2013).

There is evidence that oxidative stress causes nNOSμ relocation from the sarcolemma as well as dephosphorylation of FOXO3a transcription factor as an early event during the mechanical unloading. The redox signaling serves here as a signal for nNOS to change morphology of skeletal muscle fibers. This was proven by using EUK-134, a cell-permeable mimetic of superoxide dismutase and catalase. This agent enabled examination of the redox signaling in nNOSμ translocation and atrophy of muscle fibers upon short-term (54 h) limb unloading (Lawler et al., 2014). Interestingly, terrestrial gravity force may play an effect in the regulation of NOS relocation. For instance, the effect of microgravity on skeletal muscle was examined in rat and mice after short-term (5–20 days) spaceflights. Spaceflight induces translocation of sarcolemmal nitric oxide synthase-1 (NOS1) into the cytosol in soleus but not in the fast-twitch extensor digitorum longus muscle (Sandonà et al., 2012).

In Duchenne muscular dystrophy (DMD) patients and murine mdx model of DMD, dystrophin deficiency causes a decrease in muscle and mislocalization of muscle specific isoform nNOSμ leading to dysfunction. Systematic study of Uaesoontrachoon et al. demonstrates the effects of a NO-donating naproxen derivative, naproxcinod, on skeletal and cardiac disease phenotype in mdx mice subjected to treadmill sessions twice weekly with functional and behavioral assessments at 3, 6, and 9 months of treatment. Naproxcinod treatment significantly improves hind limb grip strength and results in less inflammation (Uaesoontrachoon et al., 2014).

These data may suggest that NO, whose production is increased during physical exercise through various pathways, is involved in the correction of functional disorders. This occurs rather through modulation of vascular function than through a direct impact on glucose utilization by skeletal myocytes. Cardiovascular patients greatly benefit from the administration of moderate physical exercise. We hypothesize that therapeutic effects of physical training can be associated with NO production. In available literature, there is lack of direct evidence in favor of this hypothesis. However, there is plenty of indirect data supporting this idea. Though it remains an open question, we would like to present this hypothesis to stimulate further studies and discussion.

## Conclusions

**Figure 1:**
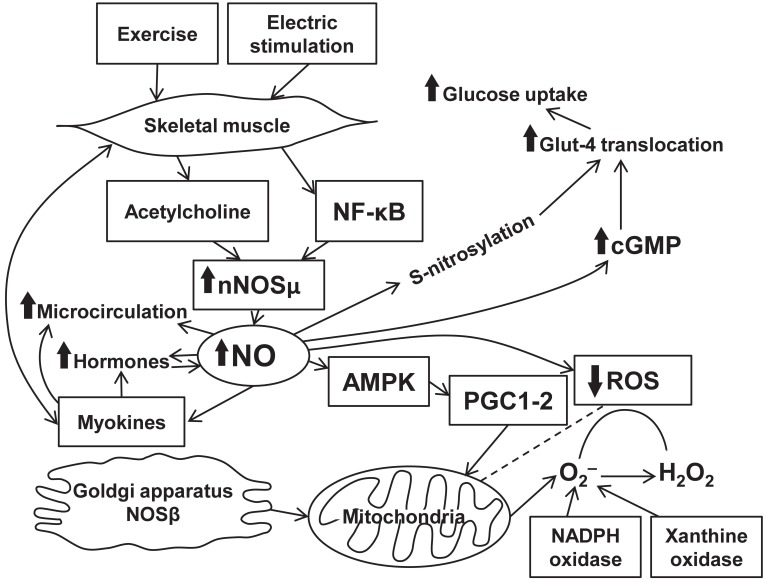
**Interplay between exercise, NO production, and signaling pathways in skeletal muscle**.

Nitric oxide production is intrinsically associated with physical activity. Enhanced NO release in physical exercise is mediated through eNOS gene transcription. Effects of NO release on muscle activity are mostly beneficial and occur through various signaling pathways (Figure 1). The mechanisms comprise well-known abilities of NO to cause vasodilation (Black et al., 2008), enhance regional blood flow/microcirculation, and to modulate hormone-dependent energy supply of muscle contraction. At the same time, NO directly affects myocytes and increases energy production in the mitochondria. Scarce data suggest the presence of the associations between NO- and myokine-dependent signaling mechanisms exerting various beneficial physiological effects. However, these effects of NO are limited and, in certain situations, can turn into the opposite leading to free radical accumulation and mitochondrial fragmentation. Evidence confirms an important role of NO in various diseases. Indeed, diabetes is associated with an increase in eNOS expression upregulated during exercise. In patients with chronic heart failure, beneficial effects of physical training are caused by impact of NO on the skeletal muscle energy metabolism associated with NO production (Okutsu et al., 2014). There is a positive feedback between NO-dependent production of certain myokines and angiogenesis that occurs in the presence of ischemia. Beneficial effects of NO are documented in degenerative muscle diseases. Nitric oxide-mediated signaling pathway clearly represents a promising target for a treatment of a variety of diseases and for achievement of better athletic performance. All in all, skeletal muscle produces plenty of bioactive substances including NO which plays a key role in a readjustment of all autonomic systems of the organism during exercise.

### Conflict of interest statement

The authors declare that the research was conducted in the absence of any commercial or financial relationships that could be construed as a potential conflict of interest.
